# Supplementary material for: A unified rheological model for cells and cellularised materials
Source: R Soc Open Sci. 2020 Jan 22;7(1):190920. doi: 10.1098/rsos.190920 (PMC7029884; doi:10.1098/rsos.190920)
Supplement: Supplementary materials [file rsos190920supp1.pdf]

Supplementary materials of "A unified rheological model for cells and cellularised materials"

A Bonfanti, J Fouchard, N Khalilgharibi, G Charras, A Kabla

1 Mechanical testing of suspended epithelial monolayers

Experiments were performed on suspended MDCK II epithelial monolayers generated as described in [1]. Cells stably expressing E-Cadherin-GFP were used. After digestion of collagen substrate, monolayers were tested at room temperature in Leibovitz's L15 medium (Gibco, ThermoFisher) supplemented with 10% FBS (Sigma).

Relaxation experiments were performed as described in [2]. Briefly, a mechanical testing setup assembled on top of an inverted microscope (Olympus IX-71) was used. First, the Petri dish containing the stretcher device was secured to the microscope stage. Next, an opto-mechanical force transducer (SI-KG7A, WPI) with a tweezer-shaped mounting hook (SI-TM5-KG7A-97902, WPI) was mounted on a motorized translation stage (M126-DG1, Physike Instrumente). The tip of the mounting hook was then brought into contact with the flexible arm of the stretcher device. The monolayer, suspended between the two arms of the device, was stretched by moving the flexible arm away from the rigid arm, using the motorized stage. Monolayers were extended to 30% strain at a rate of 75%/s. The deformation was then maintained constant during stress relaxation. The force applied to the monolayer was calculated by subtracting the force applied by the flexible arm from the total measured force. Ramp experiments were conducted on the same setup and monolayers were extended at a rate of 1%/s. Force measurements in both tests was acquired at 6.7 Hz.

For creep experiments, the setup was slightly adapted (figure S1). The deformation was controlled by moving the rigid arm connected to one extremity of the monolayer while the force was measured on the other static arm. Monolayers were prestretched by 10% prior to experiments and left to rest for 5 minutes before starting testing. Then, an initial stretch step was applied at 1.5 mm/s (about 100%/s) and the force reached was clamped using a feedback algorithm (Labview, National Instruments) and updated at 7.5 Hz.

To monitor the shape of the tissue and ensure its integrity during the tests, bright-field images were taken at a rate of 1 image/s using a 2X objective (2X PLN, Olympus) and a CCD camera (PointGrey, Grasshopper 3).

2 The fractional element: springpot

The spring-pot rheological element exploits the mathematical concept of fractional derivative to model behaviours that are intermediate between a spring and a dashpot (see figure S2). Different definitions of fractional differentiation are available. Here we use the Caputo's derivative definition; it has shown a better applicability to real problems, where initial conditions are known in terms of derivative of integer index [3]. If we assume that the system is at rest for time $t \leq 0$, the fractional derivative is given by [4]

$$\frac{d^{\beta}\epsilon(t)}{dt^{\beta}} = \frac{1}{\Gamma(1-\beta)}\int_0^t (t-\bar{t})^{-\beta}\frac{d\epsilon(\bar{t})}{d\bar{t}}d\bar{t}, \tag{S1}$$

where $\Gamma(\cdot)$ is the Gamma function and $0 < \beta < 1$. Note that in order to get the value of the derivative at a given time t, it is necessary to integrate from $t = 0$. Thus, the response of the springpot is determined by its whole deformation history. This highlights the hereditary feature of such an element.

From a graphical representation of the linear viscoelastic model as a network of springs, dashpots and springpots, we can establish the time-dependent differential equation describing the relation between stress and strain. For the model presented here the temporal evolution is given by [5]

$$\sigma(t) + \frac{\eta}{c_\beta}\frac{\mathrm{d}^{1-\beta}\sigma(t)}{\mathrm{d}t^{1-\beta}} = \eta\frac{\mathrm{d}\epsilon(t)}{\mathrm{d}t} + k\epsilon(t) + \frac{\eta k}{c_\beta}\frac{\mathrm{d}^{1-\beta}\epsilon(t)}{\mathrm{d}t^{1-\beta}}, \tag{S2}$$

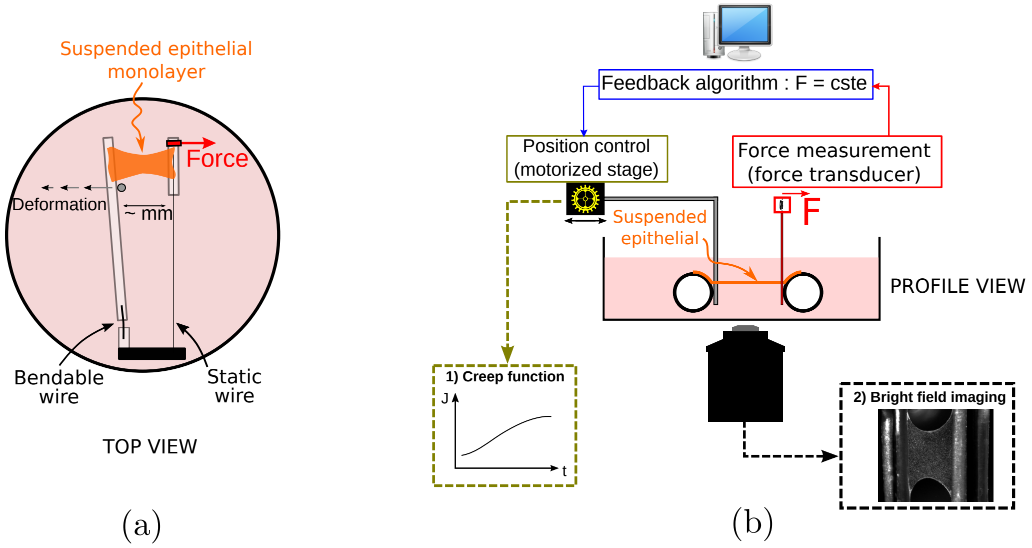

Figure S1: Set up of the creep experiment. (a) A millimetric MDCK epithelial monolayer devoid of substrate is suspended between two test rods. The device is glued to the Petri dish. The force is monitored on one side of the tissue, while the deformation is imposed on the other side. (b) A constant stress in the tissue is maintained by monitoring the traction force on the tissue through a force transducer (right) and adjusting its length in real time via a motorized stage. A PID feedback algorithm ensures the control of the stress.

where η is the viscosity of the dashpot, c_β and β are the parameters of the spring-pot and k the stiffness of the spring.

Many solutions of fractional differential equations obtained based on generalized viscoelastic models admit a closed form involving the Mittag-Leffler function [5]. Such a function is defined as

$$E_{a,b}\left(z\right) = \sum_{k=1}^{\infty} \frac{z^k}{\Gamma(ak+b)} \quad (a > 0, b > 0). \tag{S3}$$

Its qualitative behaviour for the parameters of the soft material analyzed here is plotted in figure S3.

For clarity, we briefly discuss the qualitative behaviour of the the dissipative Fractional Maxwell arm, whose relaxation modulus is given by

$$G(t) = c_\beta t^{-\beta} E_{1-\beta,1-\beta}\left(-\frac{c_\beta}{\eta} t^{1-\beta}\right), \tag{S4}$$

where c_β and β are the spring-pot parameters and η the dashpot viscosity. When β is equal to zero, then the model corresponds to a traditional Maxwell model, whose relaxation modulus assumes an exponential form (figure S4 (a), straight blue line in semi-logarithmic scale). When β increases the Fractional Maxwell model exhibits a relaxation modulus that is initially power-law, followed by a second regime where the dissipation occurs at higher rate (figure S4, black and blue curves).

3 Curve fitting and prediction

Relaxation curves collected during stress relaxation tests were analyzed using the open source library RHEOS [6] developed in *Julia* language [7].

During a typical experimental test, the strain $\epsilon(t)$ is rapidly applied at a constant rate until it reaches 30% and then maintained constant. The stress is obtain from the Boltzman integral, given by the convolution between the relaxation modulus and the derivative of the strain

$$\sigma(t) = \int_0^t G(t-\bar{t}) \frac{d\epsilon(\bar{t})}{d\bar{t}} d\bar{t}. \tag{S5}$$

where $G(t)$ is the relaxation modulus which takes the form reported in equation (2.1) in the manuscript.

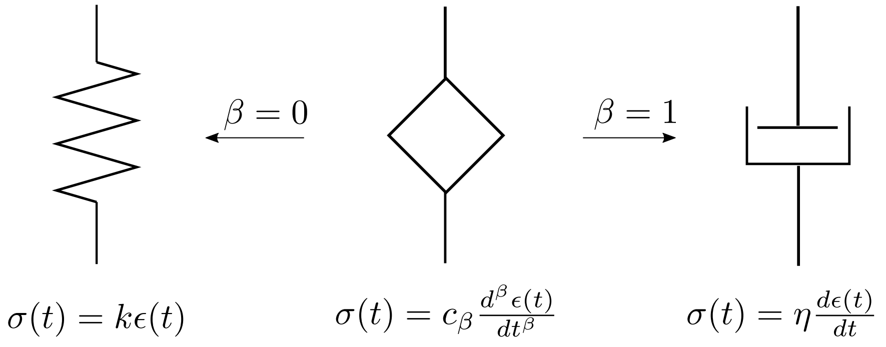

(a)

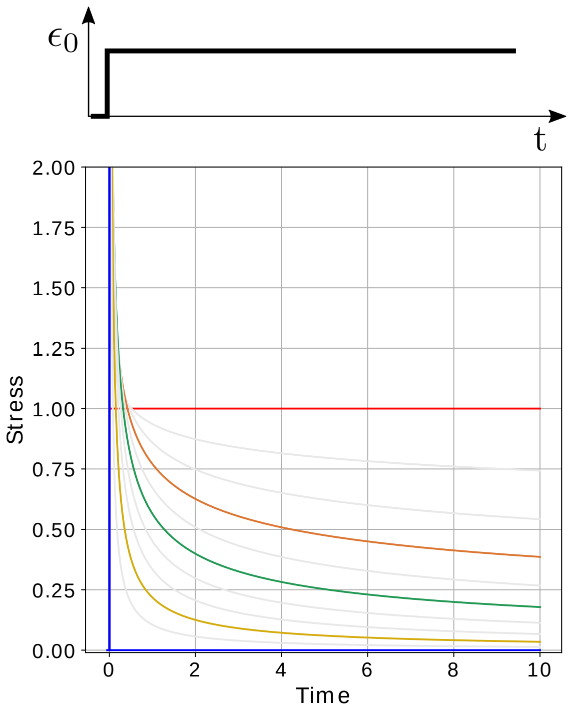

(b)

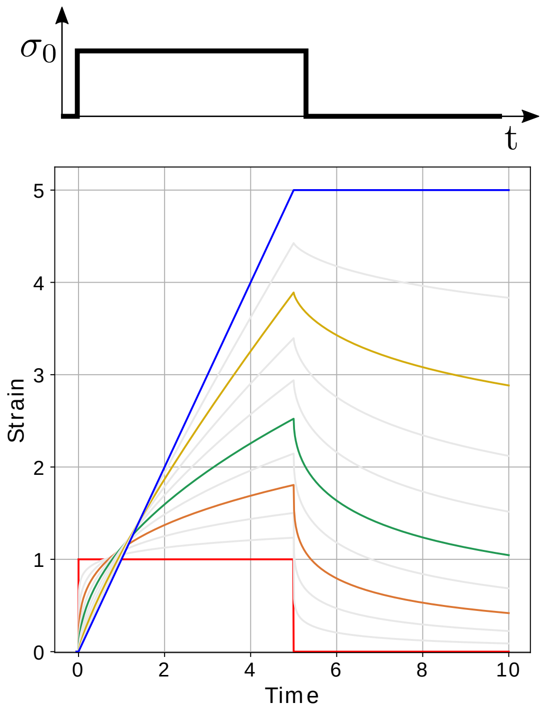

(c)

Figure S2: (a) Sketch of the fractional element–spring-pot. The spring-pot interpolates between a spring and a dashpot. When $\beta = 0$, the spring-pot reduces to a spring; whilst when $\beta = 1$ it becomes a dashpot. Consequently, the material constant c_β represents respectively the elastic constant of a spring, k (Pa) and the viscosity of a dashpot, η (Pa s). (b) Responses of a springpot when subjected to a step in stain and (c) to a step in stress. The red curves are for a spring ($\beta = 0$), the grey curves are for increasing values of β from 0.1 to 0.9 (where orange is 0.3, green, 0.5, yellow 0.8) and the blue curves are for a dashpot ($\beta = 1$).

The viscoelastic model presented here is consistent with the empirical function introduced in [2]

$$\sigma(t) = At^{-\alpha} e^{-\frac{t}{\tau}} + B, \tag{S6}$$

where B is the residual stress, α is the power exponent and τ is the characteristic time. To demonstrate this, we first re-fited the relaxation response using the same fitting procedure as in [2]. Thus k is fixed at the residual stress after reaching the plateau defined as the average of the stress for 70 s $< t <$ 75 s. The parameters obtained for the viscoelastic model and the empirical expression are similar (see table S1). If k is left free during the fitting, a variation in the relaxation characteristic time is noticed between the two approaches, especially in the Y27632 parameters. This discrepancy could be associated to the lack of a clear plateau in the experimental time-window. For the analysis presented in this work, we have used those parameters obtained by letting k free during the fitting.

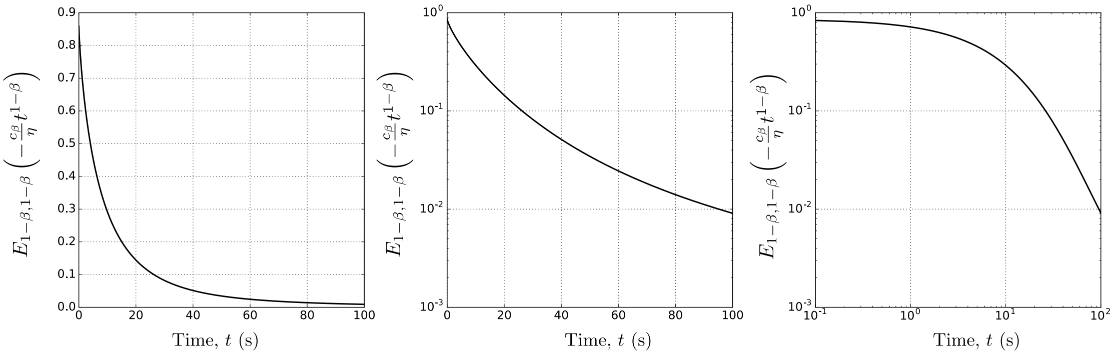

Figure S3: Mittag-Leffler function. Plot of the Mittag-Leffler function with parameters similar to those obtained for epithelial sheets ($\beta = 0.22$, $c_\beta = 1.3 \times 10^3$ Pa s$^\beta$, $\eta = 1.0 \times 10^4$ Pa s) in (a) linear, (b) semilogarithmic and (c) logarithmic scales.

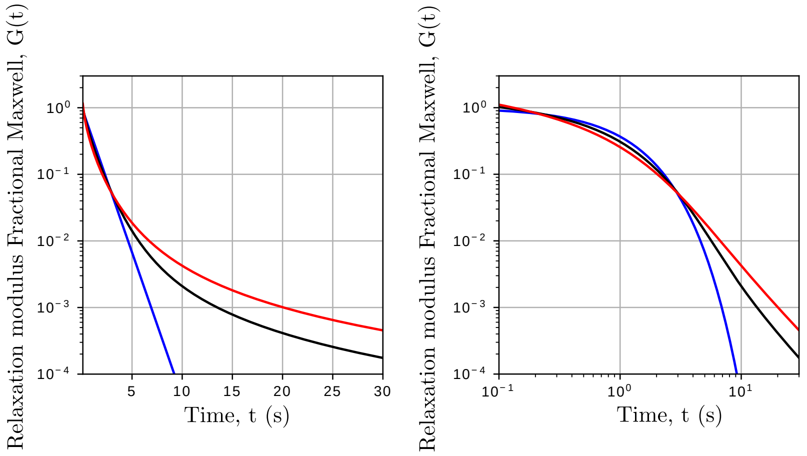

(a) (b)

Figure S4: Relaxation modulus of the Fractional Maxwell model (dissipating branch) in (a) semi-logarithmic scale and (b) log-log scale. c_β and η have been set to one. The blue curve is the relaxation modulus of the Maxwell model, $\beta = 0$, which shows an exponential behaviour at all time (straight line in the semi-logarithmic scale); the red curve is the modulus for $\beta = 0.2$, a typical value of the exponent for epithelial monolayers; the black curve is for an intermediate value of $\beta = 0.1$.

Table S1: Comparison within the parameters β power exponent, k stiffness of the spring and τ characteristic relaxation time obtained from the fitting of: (a) the empirical function in [2], (b) the model presented here when k is fixed, as done in [2] and (c) when k is free.

Sample		Control	DMSO	Y27362	CK666	SMIFH2
β	Empirical	0.28 ± 0.02	0.29 ± 0.03	0.26 ± 0.05	0.31 ± 0.01	0.29 ± 0.02
	k fixed	0.30 ± 0.03	0.27 ± 0.02	0.287 ± 0.01	0.29 ± 0.02	0.28 ± 0.02
	k free	0.28 ± 0.03	0.27 ± 0.02	0.296 ± 0.05	0.30 ± 0.02	0.29 ± 0.03
k (Pa)	Empirical	820 ± 373	1130 ± 327	303 ± 97	1290 ± 340	1213 ± 323
	k fixed	850 ± 364	1270 ± 492	300 ± 101	1286 ± 340	1212 ± 321
	k free	840 ± 376	1200 ± 428	187 ± 101	1290 ± 337	1194 ± 348
τ (s)	Empirical	11.3 ± 3.8	13.3 ± 5.5	23.3 ± 7.4	16.8 ± 6.2	19.5 ± 3.5
	k fixed	17.2 ± 7.1	11.8 ± 4.31	24.2 ± 12.7	20.42 ± 13	21.2 ± 11.5
	k free	20 ± 15.2	13.58 ± 11.6	213.83 ± 246	19.51 ± 14	34.27 ± 34

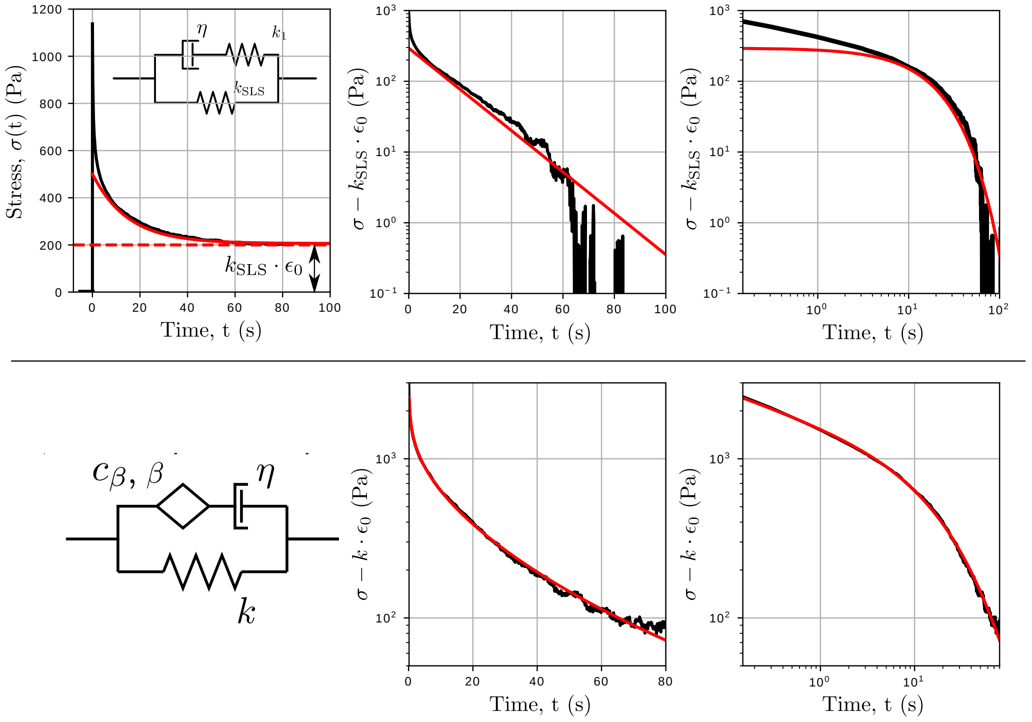

Figure S5: Comparison of the quality of fit between the Standard Linear Solid model (upper row) and the fractional viscoelastic model introduced here (bottom row). The fits are plotted in both semi-logarithmic and log-log scale, and in lin-lin scale for the Standar linear Solid model.

To predict the response to a slow ramp, the Boltzman integral reported in equation S5 is used. The relaxation modulus $G(t)$ takes the form reported in equation (2.1) in the manuscript, while the derivative of the strain will be equal to the applied strain rate. Note that the parameters of the relaxation modulus are those predicted from the fitting of the relaxation experiments.

The prediction of the creep response is obtained by solving the Boltzman integral

$$\epsilon(t) = \int_0^t J(t - \bar{t}) \frac{d\sigma(\bar{t})}{d\bar{t}} d\bar{t}. \tag{S7}$$

where $J(t)$ is the creep modulus. To calculate the creep modulus we use the same parameters as extracted from the fitting of the relaxation experiments. The predicted creep response for the treated monolayers Y27632 is reported in figure S6 in the Supplementary materials.

4 Pharmacological treatment of monolayers

assess the generality of the fractional viscoelastic model, we analysed data resulting from a range of relevant pharmacological treatments on the MDCK monolayers as recently presented in [2]. All drugs are dissolved in Dimethylsulfoxide (DMSO), and monolayers treated with DMSO alone were used as a control to assess the effect of each drug. Y27632 has a significant effect on the materials rheology, as discussed in the main paper. Treatment with CK666, that prevents actin polymerisation through Arp2/3, does not show any effect of the treatment on the relaxation response. Monolayers treated with SMIFH2, that prevents formins-mediated actin polymerisation, revealed that formin inhibition slightly increases the relaxation characteristic time (see table S1); we can attribute such increment to a higher viscosity η, whilst all the other parameters remain almost unchanged S7. Similarly, the two most abundant actin crosslinkers identified in previous RNAseq experiments [2] were perturbed. The inhibition of either F-actin crosslinkers filamin A (FLNAShRNA) or α-actinin 4 (ACTNShRNA) do not affect stress dissipation S8, suggesting that crosslinkers do not play a role in the relaxation response. These results are broadly in agreement with the analysis presented in [2].

5 Master curve for the relaxation response

The fact that untreated and treated monolayer can be fitted with the same model, sharing similar values of the exponent β, suggests the possibility of identifying a master curve that describes all the experimental data. For this purpose, we simplify the analysis by assuming that the loading is instantaneously applied, thus the stress can be written as $\sigma(t) = G(t)\epsilon_0$, where $G(t)$ is the relaxation modulus and ϵ_0 the applied stress. By re-writing the relaxation modulus in equation 2.1 in the main manuscript in terms of the characteristic relaxation time and removing the shift introduced by the addition of a spring in parallel

$$G(t)^* = G(t) - k = c_\beta \tau_1^{-\beta} \left(\frac{t}{\tau_1}\right)^{-\beta} E_{1-\beta,1-\beta}\left(-\left(\frac{t}{\tau_1}\right)^{1-\beta}\right), \tag{S8}$$

we can identify the additional non-dimensionalising factor for the relaxation modulus k_{eff}

$$k_{\mathrm{eff}} = c_\beta \tau_1^{-\beta} = \left(\frac{c_\beta}{\eta^\beta}\right)^{1/(1-\beta)}, \tag{S9}$$

which represents the *effective* stiffness of the Fractional Maxwell Model (FMM), the dissipative branch. Note that τ_1 can be also derived from the definition of the characteristic relaxation time for the Maxwell model as $\tau_1 = \eta/k_{\mathrm{eff}}$. τ_1 is independent from the spring stiffness since the stiffness k only shifts the stress towards higher values, as seen in equation 2.1.

The nondimensionalized relaxation modulus $\overline{G}(t)^* = (G(t)-k)/k_{\mathrm{eff}}$ is now a function of only two parameters $\overline{G}(t)^* = g(t/\tau_1, \beta)$, where the time has been nondimensionalized using the characteristic relaxation time τ_1, while the relaxation modulus has been normalized using the effective stiffness k_{eff}. Consistent with the observation that their short time-scale response is comparable, after rescaling the relaxation curves in figure 2 (b)-(c), all the curves collapse onto one master curve (see figure S9 (a)). From the fitting of the relaxation responses of treated monolayers, we observe that β remains constant. Therefore, the nondimensional relaxation modulus can be considered a function of only one parameter $\overline{G}(t)^* = g(t/\tau_1)$, because β remains constant. Because of this, all the responses obtained for the other treatments collapse to one master curve (figure S9 (b)).

6 Statistical analysis

Statistical analysis of the data was performed in *Julia* language using a Wilcoxon rank-sum test. Changes with statistical power greater than 0.01 were considered non-significant (ns). Dataset different $p<$ 0.01 are denoted by an asterisk (*).

The material parameters measured from the relaxation experiment show some variability. We assume that they follow a Gaussian distribution. To take this into account, we take an interval for each of the parameters equal to Mean $\pm 1.96\sigma/\sqrt{n}$, where σ here is the standard deviation and n the number of samples. The colored areas within the two curves obtained by taking lower and upper bounds of the parameters is where the predicted response of the material will lay.

7 Mechanical testing of single epithelial cells

Experiments were performed on MDCK II epithelial cells previously trypsinised and plated in a Petri dish and left to settle for 10-30 minutes. Before cells started to spread, they were tested while still in rounded shape.

Relaxation experiments were performed as described in [2]. A CellHesion 200 Atomic Force Microscope was mounted on a scanning laser confocal microscope. Tipless silicon SPM-Sensor cantilevers with nominal spring constant of 0.03N m^{-1} were used to perform the experiments. The cantilever spring constant for each experiment was calibrated using the thermal noise fluctuation method and it ranged between 0.055-0.06 N m^{-1}.

The target force required to indent the cell by $\sim$ 30% was estimated by approximating the cell height. The latter was given by the difference between the force-displacement curves acquired on the cell and a glass region close to it. Cells were subjected to the target force of 5-40 nN at a rate of 75% s^{-1}. The cantilever beam was maintained at constant height for 150 s while the evolution of the force was recorded.

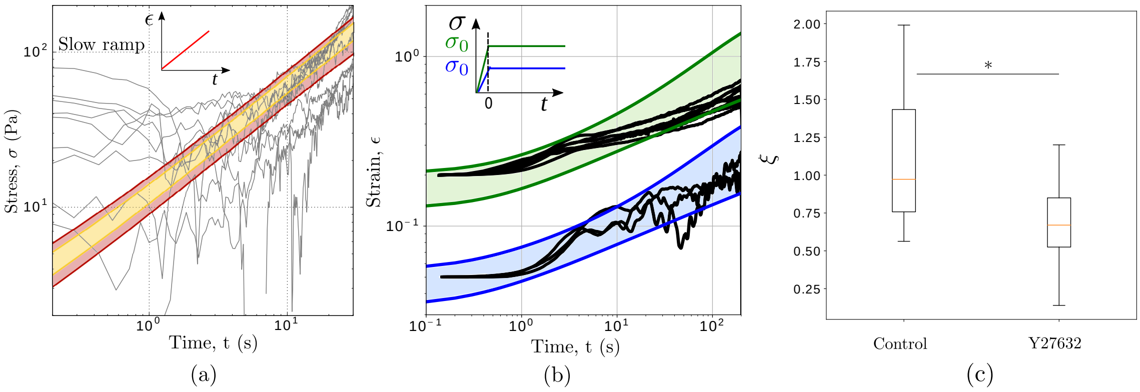

Figure S6: (a) Predicted stress response of the Y27632 treated monolayers when subjected to a slow stretch (1%/s) using the mechanical parametrization determined from stress relaxation experiments. The predicted responses (95% confidence interval red areas and 70% yellow areas) are in good agreement with the experimental data (black curves). (b) The creep response of epithelial monolayers in which actomyosin contractility is inhibited. The creep is performed at 60 Pa (blue area) and 230 Pa (green area). The black lines are the experimental results. (c) ξ value for untreated and treated monolayers which give raise to different qualitative behaviours ($p < 0.01$).

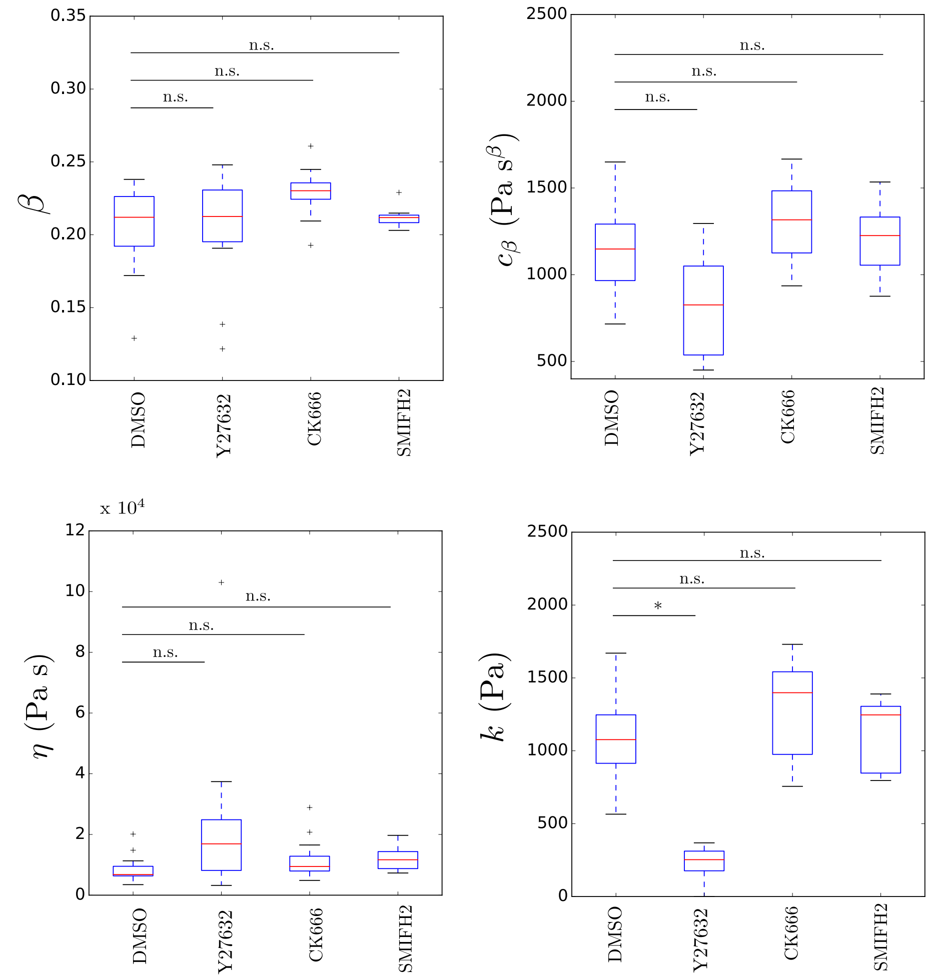

Figure S7: Boxplots comparing the model parameters of monolayers subjected to chemical treatments targeting the actomyosin cytoskeleton. β: $p = 0.64$ for Y27632, $p = 0.019$ for CK666, $p = 0.98$ for SMIFH2, c_β: $p = 0.015$ for Y27632, $p = 0.053$ for CK666, $p = 0.50$ for SMIFH2, η: $p = 0.04$ for Y27632, $p = 0.053$ for CK666, $p = 0.022$ for SMIFH2, k: $p < 0.01$ for Y27632, $p = 0.19$ for CK666, $p = 0.60$ for SMIFH2, for all compared to DMSO.

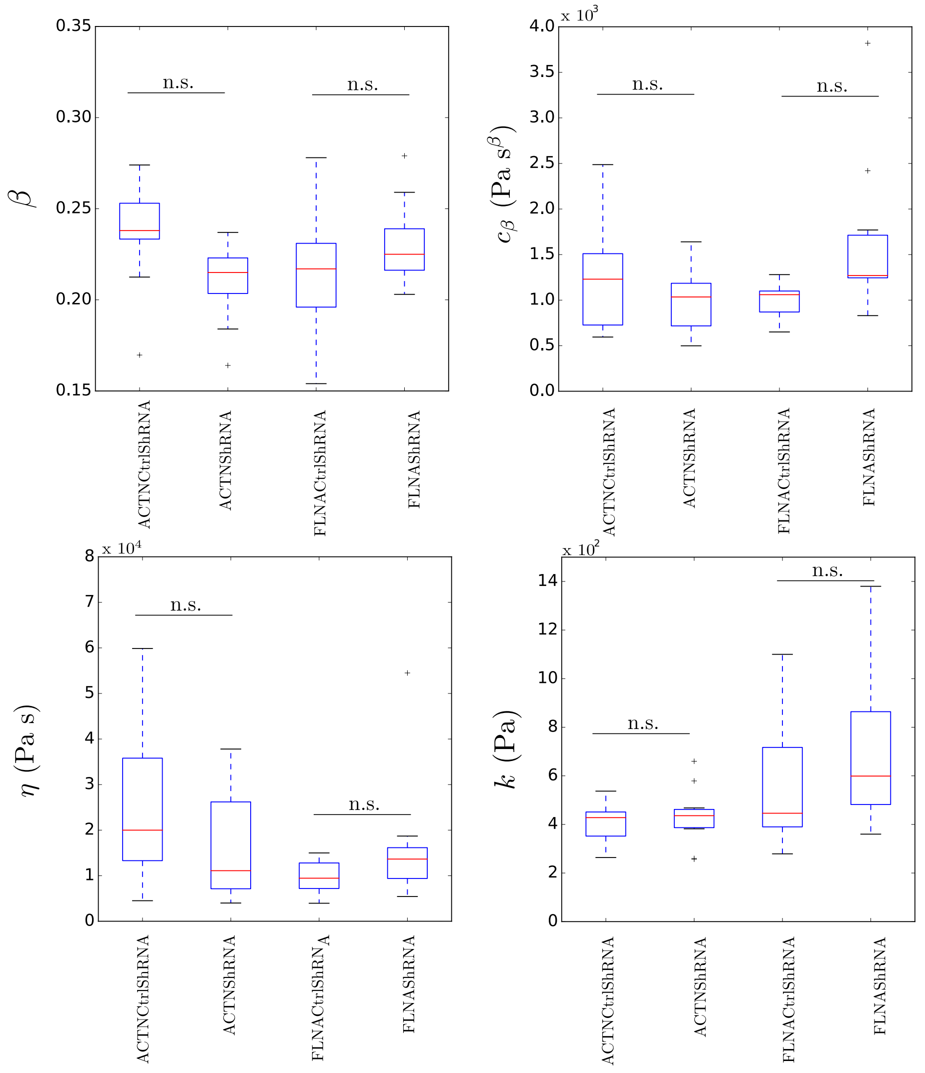

Figure S8: Boxplots comparing the model parameters of monolayers in which the F-actin crosslinkers filamin A (FLNAShRNA) or α-actinin 4 (ACTNShRNA) were perturbed. β: $p = 0.03$ for ACTNShRNA and $p = 0.18$ for FLNAShRNA, c_β: $p = 0.71$ for ACTNShRNA and $p = 0.018$ for FLNAShRNA, η: $p = 0.28$ for ACTNShRNA and $p = 0.09$ for FLNAShRNA, k: $p = 0.78$ for ACTNShRNA and $p = 0.25$ for FLNAShRNA, compared to their respective controls, ACTNCtrlShRNA and FLNACtrlShRNA.

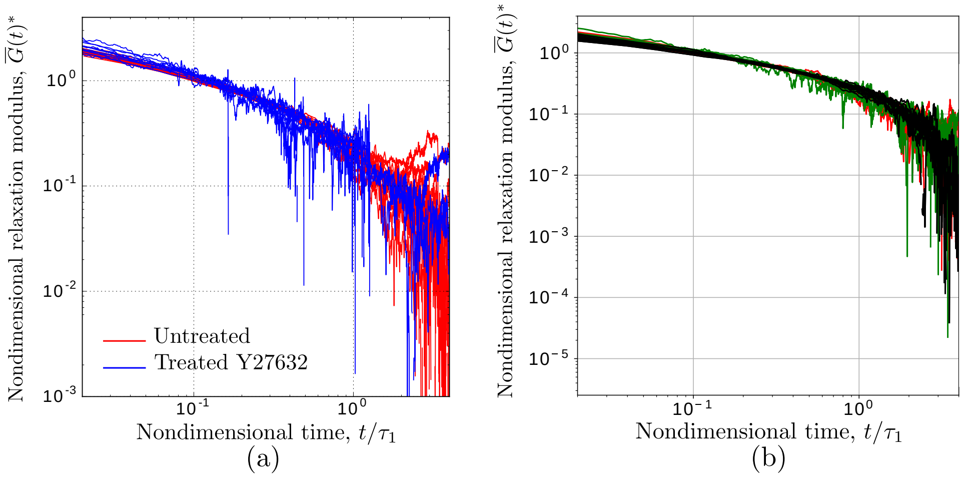

Figure S9: Master curve for the relaxation response. (a) Experimental relaxation curves scaled using the characteristic time τ_1 and the effective stiffness of the Fractional Maxwell Model k_{eff}. The red curves are the experimental data for the untreated monolayers, while the blue curves are the experimental data for the Y27632 treated monolayers. All the data collapse onto a master curve demonstrating that they possess a similar value of β. (b) Scaling of the relaxation responses for the other treatments: black DMSO, red CK666, green SMIFH2. All curves collapse to one master curve since they possess a similar value of β, the springpot exponent.

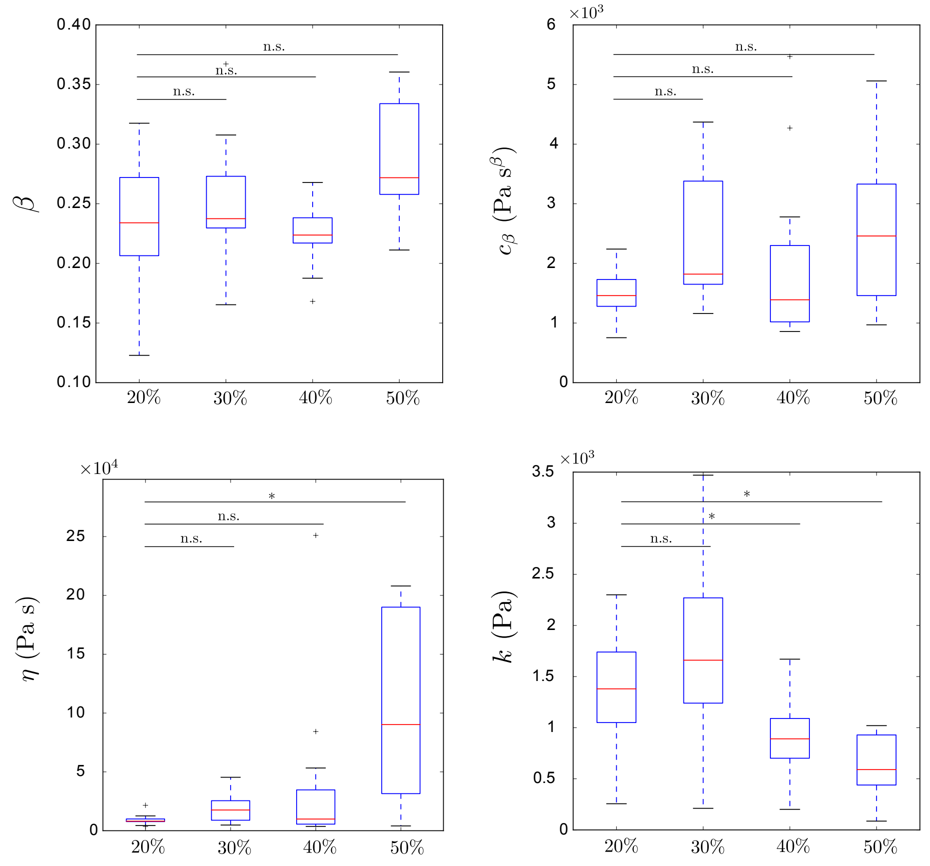

Figure S10: Test of linearity. The average parameters obtained from the fitting of the relaxation curves for tissues subjected to different strain amplitudes (samples' number for each value of strain is 13): (a) power-law exponent β (p = 0.51 for 30%, p = 0.51 for 40%, p = 0.03 for 50%). (b) spring-pot coefficient c_β (p = 0.03 for 30%, p = 0.90 for 40%, p = 0.04 for 50%) (c) viscosity η (p = 0.05 for 30%, p = 0.29 for 40%, p = 0.0012 for 50%) (d) stiffness k (p = 0.44 for 30%, p = 0.007 for 40%, $p < 0.001$ for 50%).

Table S2: Fitted parameters.

Sample	η (Pa s)	β	c_β (Pa s$^\beta$)	k (Pa)	τ_1 (s)	τ_2 (s)	ξ
Epithelial MDCK monolayer [2]	$1 \times 10^4 \pm 8 \times 10^3$	0.22 ± 0.02	1360 ± 420	760 ± 340	13	13	1
Epithelial MDCK monolayer, Y27632 treated [2]	$5 \times 10^4 \pm 2 \times 10^4$	0.28 ± 0.05	1120 ± 441	187 ± 100	195	270	0.7
Single epithelial MDCK cell [2]	870 ± 720	0.14 ± 0.03	109 ± 20	203 ± 32	10	4	2.6
Single articular chondrocyte [8]	∞	0.70	140	133	∞	∞	∞
Collagen fibrils [9]	722 (MPa s)	0.14	14 (MPa s$^\beta$)	83 (MPa)	100	10	11
Single C2-7 myogenic cells [10]	4×10^5	0.26	885	0.0	∞	-	-
Cytoplasm [11]	35	0.3	0.7	0.16	270	218	1
Yolk cell [11]	16×10^3	0.15	0.62	0.52	∞	-	-
HeLa Kyoto cell cortex [12]	170 (mN/m s)	0.22	48 (mN/m s$^\beta$)	5 (mN/m)	-	-	-
Human primary immune cells [13].	∞	0.26	600	0.0	-	-	-

8 The complex modulus of the novel generalized fractional model

Although the relaxation modulus $G(t)$ and the creep compliance $J(t)$ each fully summarize the viscoelastic linear response of a material, the time-dependent response is often characterized using the complex elastic modulus, which captures the ratio and phase difference between stress and strain under oscillatory deformation, which is particularly relevant to biomaterials because these are often subjected to cyclical stresses. The complex modulus can be decomposed into a real $G'(\omega)$ and an imaginary $G''(\omega)$ part, which represent respectively the storage modulus that accounts for the elastic response, and the loss modulus that accounts for the dissipative response. The complex modulus of the viscoelastic model presented here can be derived from the Laplace transform of the creep compliance $J(s)$ and is given by:

$$G'(\omega) + \mathrm{i}G''(\omega) = \frac{k\left[\mathrm{i}\omega\tau_2 + 1 + (\mathrm{i}\omega\tau_1)^{1-\beta}\right]}{1 + (\mathrm{i}\omega\tau_1)^{1-\beta}}. \tag{S10}$$

By evaluating the real and imaginary parts of the equation above, we find that the storage and loss moduli are respectively given by

$$G^{'}(\omega) = k\left[\frac{(\omega\tau_1)^{1-\beta}(\omega\tau_2)\sin\left((1-\beta)\frac{\pi}{2}\right)}{1 + (\omega\tau_1)^{1-\beta}\left[(\omega\tau_1)^{1-\beta} + 2\cos\left((1-\beta)\frac{\pi}{2}\right)\right]} + 1\right] \tag{S11}$$

and

$$G^{''}(\omega) = k\frac{(\omega\tau_2)\left[1 + (\omega\tau_1)^{1-\beta}\cos\left((1-\beta)\frac{\pi}{2}\right)\right]}{1 + (\omega\tau_1)^{1-\beta}\left[(\omega\tau_1)^{1-\beta} + 2\cos\left((1-\beta)\frac{\pi}{2}\right)\right]}. \tag{S12}$$

Storage and loss moduli predicted for the monolayers using the parameters obtained from the relaxation experiments both show a power-law behaviour at high frequencies with the same exponent, as shown in figure S11. By contrast, at low frequencies the material exhibits solid-like behaviour [14, 15]. Two special cases can be distinguished at the upper and lower limit of β; when the two moduli reduce respectively to those of the Standard Linear Solid model and the Kelvin-Voigt model, as confirmed in figure S11 (b). By letting β vary between 0 and 1, we can observe transitory behaviours between an SLS and a Kelvin-Voigt model (see figure S12).

References

[1] Andrew R Harris, Julien Bellis, Nargess Khalilgharibi, Tom Wyatt, Buzz Baum, Alexandre J Kabla, and Guillaume T Charras. Generating suspended cell monolayers for mechanobiological studies. *Nature protocols*, 8(12):2516, 2013.

[2] Nargess Khalilgharibi, Jonathan Fouchard, Nina Asadipour, Ricardo Barrientos, Maria Duda, Alessandra Bonfanti, Amina Yonis, Andrew Harris, Payman Mosaffa, Yasuyuki Fujita, et al. Stress relaxation in epithelial monolayers is controlled by the actomyosin cortex. *Nature Physics*, page 1, 2019.

[3] Mi Di Paola, A Pirrotta, and A Valenza. Visco-elastic behavior through fractional calculus: an easier method for best fitting experimental results. *Mechanics of materials*, 43(12):799–806, 2011.

[4] R Gorenflo and F Mainardi. Fractional calculus: Integral and differential equations of fractional order. *Fractals and Fractional Calculus in Continuum Mechanics*, 1(1):223–276, 2008.

[5] H Schiessel, R Metzler, A Blumen, and TF Nonnenmacher. Generalized viscoelastic models: their fractional equations with solutions. *Journal of physics A: Mathematical and General*, 28(23):6567, 1995.

[6] Louis Kaplan, Alessandra Bonfanti, and Alexandre Kabla. Rheos.jl - a julia package for rheology data analysis. *Journal of Open Source Software*, 4(41):1700, 2019. https://github.com/JuliaRheology/RHEOS.jl.

[7] Jeff Bezanson, Alan Edelman, Stefan Karpinski, and Viral B Shah. Julia: A fresh approach to numerical computing. *SIAM review*, 59(1):65–98, 2017.

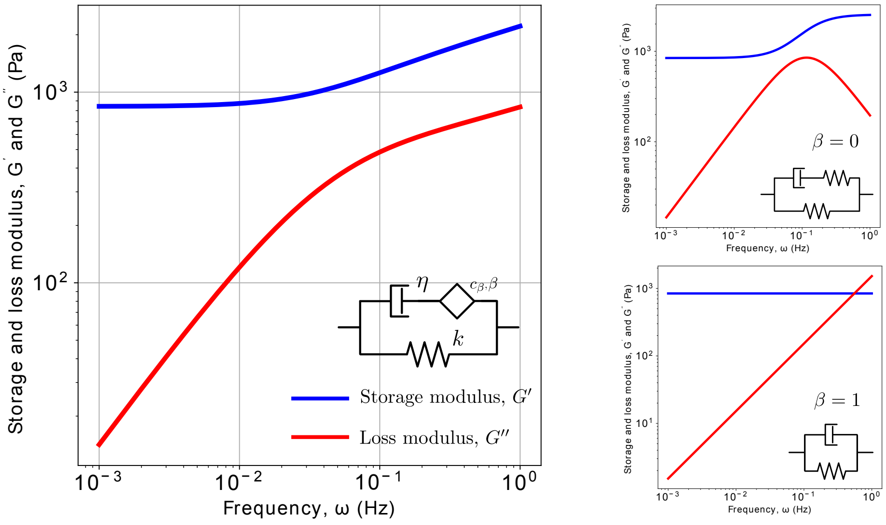

(a) (b)

Figure S11: Storage and loss moduli of the fractional model shown in figure 2 (a). (a) The blue and red lines are respectively the storage and loss modulus of the viscoelastic model introduced here using the material parameters extracted from the relaxation experiment for epithelial monolayers shown on figure 1 (a) ($\beta = 0.22$, $c_\beta = 1.3 \times 10^3$ Pa s$^\beta$, $k = 760$ Pa, $\eta = 1.4 \times 10^4$ Pa s, $\tau_1/\tau_2 = 1$). (b) Moduli for two extreme cases. When $\beta = 0$ the model behaves as a Standard Linear Solid model (top), when $\beta = 1$ the model behaves as a Kelvin mode (bottom). The other parameters have been kept constant.

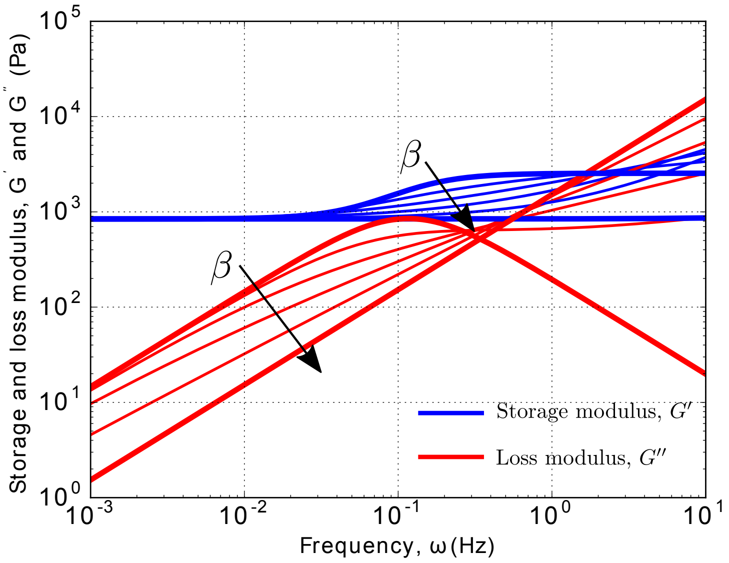

Figure S12: Intermediate behaviours from Kelvin model to Standard Linear Solid Model ($c_\beta = 1.3 \times 10^3$ Pa s$^\beta$, $k = 800$ Pa, $\eta = 1 \times 10^4$ Pa s, $\beta = 0, 0.2, 0.4, 0.6, 0.8, 1$. The blue and red lines are respectively the storage and loss moduli. Note that by changing the value of β the ratio between the two characteristic time scales changes and it is respectively given by $\tau_1/\tau_2 = 0.46, 0.85, 2.08, 12.5, 270, \infty$. The thicker curves are the behaviours of the Kelvin and Standard Linear Solid Model.

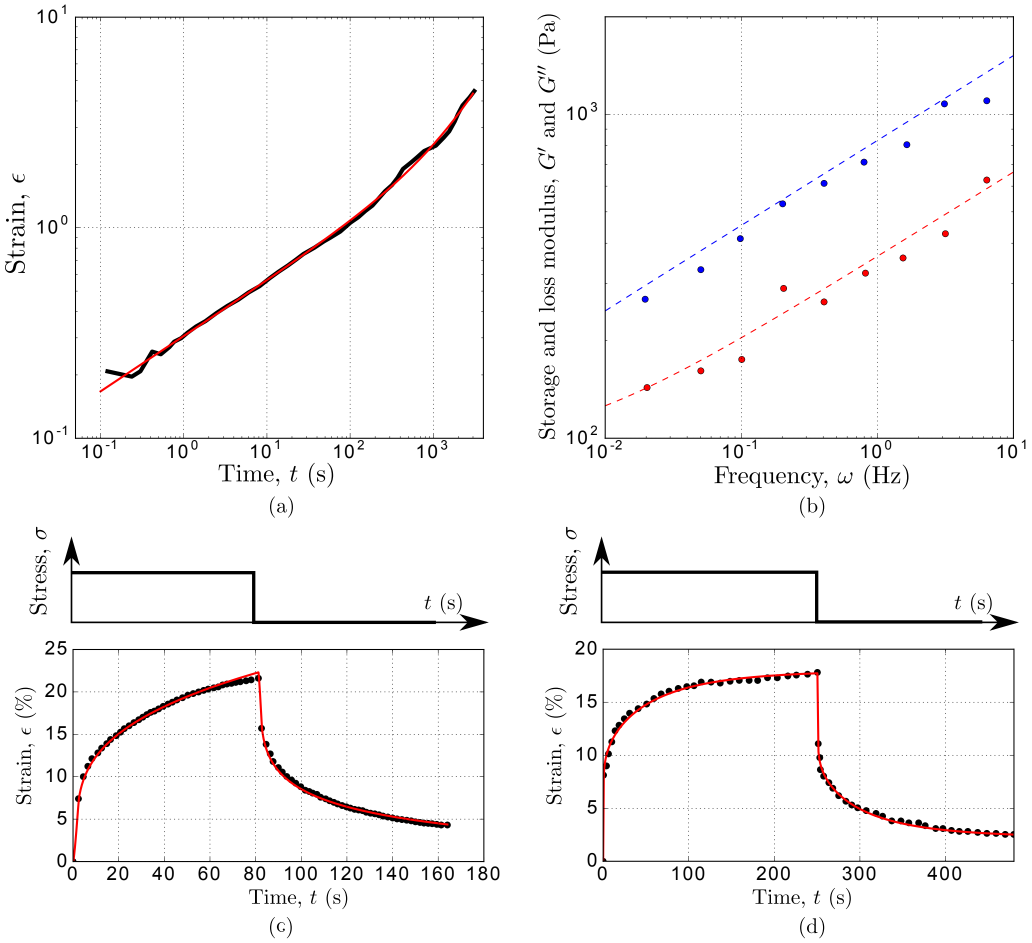

Figure S13: Examples of the application of the fractional viscoelastic model for the analysis of the mechanical response in biology. (a) Fitting of the creep response of C2-7 myogenic cells derived from skeletal muscle of adult CH3 mice (original data from [10]) with a special case of the model presented here. Fitted parameters: $\eta = 4 \times 10^5$ Pa s, $c_\beta = 885$ Pa s$^\beta$, $\beta = 0.26$ (b) The prediction of the storage and loss modulus (respectively blue and red dashed lines) of C2-7 cells using the parameters obtained from the fitting of the creep response in (a) are in good agreement with the experimental data obtained from [16] (c) Fitting of the creep and recovery response (red line) of the cytoplasm of the blastomere, and (d) the yolk cell (original data from [11], black dots) using the viscoelastic model presented here. Fitted parameters for cytoplasm: $\eta = 35$ Pa s, $c_\beta = 0.7$ Pa s$^\beta$, $\beta = 0.3$, $k = 0.156$ Pa. Fitted parameters yolk cell: $\eta = 16 \times 10^3$ Pa s, $c_\beta = 0.62$ Pa s$^\beta$, $\beta = 0.15$, $k = 0.52$ Pa.

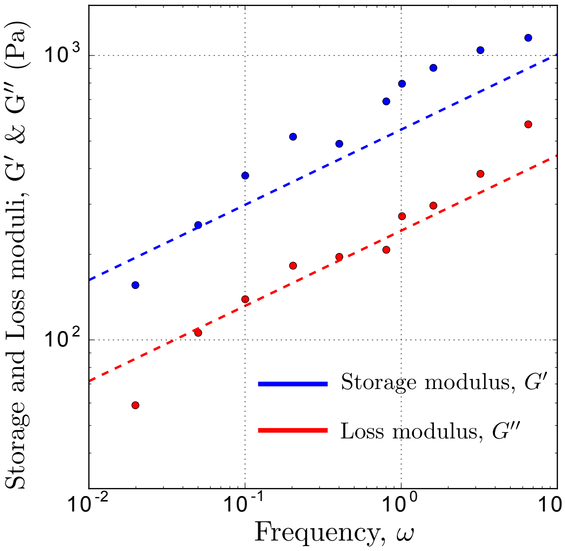

Figure S14: Fitting of storage and loss moduli from a single cell dynamic test of human primary immune cells. Original data from [13]. Fitted parameters: $\eta = \infty$ Pa s, $c_\beta = 600$ Pa s$^\beta$, $\beta = 0.26$, k $= 0$. Note that the high viscosity and the zero value of the spring are directly obtained from the fitting, without constrains on the parameters.

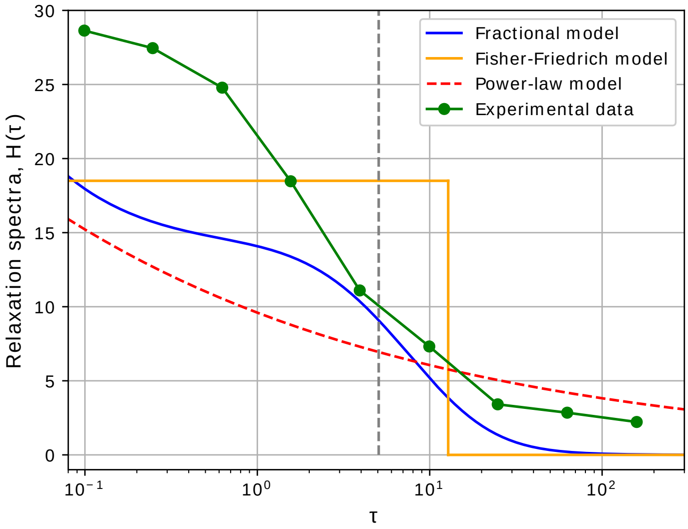

Figure S15: Comparison of the relaxation spectra of three different models with experimental data digitized from Fischer-Friedrich *et al.* [12] (green curve). The blue spectrum is calculated based on the fractional model parameters presented in figure 4 (c); the vertical grey line indicates the transition time τ_1. The orange spectrum represents the theoretical model used by Fischer-Friedrich *et al.*. The red curve corresponds to a power-law model.

[8] EM Darling, S Zauscher, and F Guilak. Viscoelastic properties of zonal articular chondrocytes measured by atomic force microscopy. *Osteoarthritis and cartilage*, 14(6):571–579, 2006.

[9] Zhilei Liu Shen, Harold Kahn, Roberto Ballarini, and Steven J Eppell. Viscoelastic properties of isolated collagen fibrils. *Biophysical journal*, 100(12):3008–3015, 2011.

[10] Nicolas Desprat, Alain Richert, Jacqueline Simeon, and Atef Asnacios. Creep function of a single living cell. *Biophysical journal*, 88(3):2224–2233, 2005.

[11] Friedhelm Serwane, Alessandro Mongera, Payam Rowghanian, David A Kealhofer, Adam A Lucio, Zachary M Hockenbery, and Otger Campàs. In vivo quantification of spatially varying mechanical properties in developing tissues. *Nature methods*, 14(2):181–186, 2017.

[12] Elisabeth Fischer-Friedrich, Yusuke Toyoda, Cedric J Cattin, Daniel J Müller, Anthony A Hyman, and Frank Jülicher. Rheology of the active cell cortex in mitosis. *Biophysical journal*, 111(3):589–600, 2016.

[13] Nathalie Bufi, Michael Saitakis, Stéphanie Dogniaux, Oscar Buschinger, Armelle Bohineust, Alain Richert, Mathieu Maurin, Claire Hivroz, and Atef Asnacios. Human primary immune cells exhibit distinct mechanical properties that are modified by inflammation. *Biophysical journal*, 108(9):2181–2190, 2015.

[14] Philip Kollmannsberger and Ben Fabry. Linear and nonlinear rheology of living cells. *Annual review of materials research*, 41:75–97, 2011.

[15] Daisuke Mizuno, Catherine Tardin, Christoph F Schmidt, and Frederik C MacKintosh. Nonequilibrium mechanics of active cytoskeletal networks. *Science*, 315(5810):370–373, 2007.

[16] N Desprat, A Guiroy, and A Asnacios. Microplates-based rheometer for a single living cell. *Review of scientific instruments*, 77(5):055111, 2006.
